# Clinical Factors Associated with COVID-19 Severity in Chronic Hospitalized Infants and Toddlers: Data from a Center in the West Part of Romania

**DOI:** 10.3390/healthcare10050808

**Published:** 2022-04-27

**Authors:** Alina Domnicu, Mirela Mogoi, Aniko Manea, Eugen Radu Boia, Marioara Boia

**Affiliations:** 1 PhD School Department, “Victor Babes” University of Medicine and Pharmacy Timisoara, Eftimie Murgu Sq., No.2, 300041 Timisoara, Romania; mitrualina@yahoo.com; 2 Pediatric Department, “Victor Babes” University of Medicine and Pharmacy Timisoara, Eftimie Murgu Sq., No.2, 300041 Timisoara, Romania; 3 Neonatology Department, “Victor Babeş” University of Medicine and Pharmacy Timisoara, Eftimie Murgu Sq., No.2, 300041 Timisoara, Romania; aniko180798@yahoo.com (A.M.); marianaboia@yahoo.com (M.B.); 4 ENT Department, “Victor Babes” University of Medicine and Pharmacy Timisoara, Eftimie Murgu Sq., No.2, 300041 Timisoara, Romania; eugen_boia@yahoo.com

**Keywords:** COVID-19, infant, toddler, risk factors, chronic diseases, malnutrition

## Abstract

Background: The risk factors for developing a severe form of COVID-19 in young children are poorly understood. Methods: A single-center retrospective study was conducted to quantify and analyze the clinical risk profile of children admitted to the Pediatric Clinic for Nutritional Recovery. Results: Overall, 51.5% (*n* = 17) of children were infected with SARS-CoV-2, all symptomatic, and five of them (29.4%) developed a severe form. A positive clinical pulmonary exam was only associated with the severe outcome (OR: 2.00; 95% CI, 0.33–5.66; *p* = 0.02). Other factors such as age under 3 months, prematurity, birth weight, malnutrition or positive history of congenital cardiac, neurodevelopmental, or genetic diseases, fever, temperature, cough, and digestive symptoms were not found to be significant risk factors. Conclusions: Clinical guidelines based on risk stratification for SARS-CoV-2 infection in children are needed in order to manage, monitor and establish priority access for some groups to high medical care.

## 1. Introduction

On 11 March 2020, the World Health Organization declared COVID-19 a global pandemic [1]. In Romania, a national state of emergency was declared 5 days later, on 16 March 2020. In the beginning, it was said that children were less affected [2]. However, in time, with every virus mutation, children of all ages became a part of the problem. In Romania, a developing East European Country, COVID-19 infection became challenging for pediatric services, especially those treating chronic patients. As of January 2022, more than 213,281 Romanian children were confirmed with SARS-CoV-2 infection, representing 10% of all cases [3]. Even though COVID-19 in children is generally asymptomatic or mild, severe outcomes are possible, and 35 of the infected children died, all known to have underlying medical conditions [3].

The risk factors for developing a severe form of COVID-19 infection at pediatric age are poorly understood. Factors such as age (1 to 3 months age or 15–18 years old), male sex, preexisting chronic or acute medical conditions (asthma, acute pneumonia), and symptom duration have been cited in various studies [4,5,6]. The unique characteristics of the immune system in children, both innate immunity and trained immunity, seem to play a role in disease susceptibility and evolution, but there are still several unanswered questions [7].

The presented study aimed to quantify and analyze the risk factors for severe outcomes in SARS-CoV-2-positive children admitted to the Pediatric Clinic for Nutritional Recovery. As Romania is confronting the fifth wave, a second aim was to develop a local protocol on the basis of clinical decisions for selecting the cases considered at high risk for developing severe forms and transferring them promptly to another pediatric ward treating acute patients.

## 2. Materials and Methods

### 2.1. Study Design

A single-center, retrospective observational study, including all patients admitted in March 2021, was conducted at the Pediatric Clinic for Nutritional Recovery of the Emergency Children Hospital “Louis Turcanu” Timisoara, Romania. The study protocol followed the Helsinki Declaration, the database was fully anonymized, and the Ethics Committee of Emergency Children Hospital “Louis Turcanu” Timisoara approved the use of preexisting clinical data (no. 19762/20 December 2021).

### 2.2. Participants

The inclusion criteria were all children admitted to the Pediatric Clinic for Nutritional Recovery during the local COVID-19 outbreak (7 March 2021). After confirming the first case of COVID-19, no new admissions were made. Those with incomplete data were excluded.

The Pediatric Clinic for Nutritional Recovery is a ward dedicated to chronic care of children under 2 years of age, suffering from malnutrition and other severe diseases (neurological, cardiac malformations, genetic syndromes, etc.), who cannot benefit from adequate care at home. This pediatric clinic is the only medical service of this type in the western region of Romania, where 50 children can be admitted simultaneously, without their parents. The parents can visit the children during visiting hours, wearing specific medical equipment. The clinic is a separate wing from the hospital’s main building. All multidisciplinary medical evaluations were conducted in the main building.

In March 2021, there was a COVID-19 outbreak, and, at the time, the infrastructure did not allow all COVID-19-positive children to be transferred to another COVID-19-dedicated ward or for acute pediatric care.

### 2.3. Study Variables, Measurements, and Definitions

Data collected from all electronic medical records were age (months), sex, gestational age (weeks), birth weight (grams, g), actual weight (g), birth length (centimeters, cm), actual length (cm), underlying medical conditions (comorbidities), and the individual vaccination schedule.

COVID-19 diagnosis was confirmed through the detection of nucleic acids by real-time polymerase chain reaction (RT-PCR) from a nasopharyngeal swab as recommended by the National Center for Surveillance and Control for Communicable Diseases Romania. The children were tested because of suspected acute infection or exposure to a confirmed case. Clinical presentation, evolution, and the type of treatment received were noted. According to hospital local guidelines, the children were retested at symptom remission or after 14 days from the first positive test. The duration until a negative RT-PCR test result was estimated.

Prematurity was defined as gestational age before 37 complete weeks. The corrected gestational age was calculated using the following formula: 40 weeks minus gestational age at birth [8,9]. Weight, length, and weight-for-length z-scores were calculated on the basis of WHO growth charts [8] or Fenton growth charts when appropriate [10,11]. Children were divided into three categories: small for gestational age (SGA), appropriate for gestational age (AGA), and large for gestational age (LGA) [8,9,10,11]. SGA is defined as birth weight and/or length less than −2.0 standard deviations (SDs) or below the 10th percentile, while LGA is defined as birth weight and/or length greater than +2.0 SDs or above the 90th percentile [9].

Undernutrition was classified into stunning (below −2.0 SDs in height for age), wasting (lower than −2.0 SDs in weight for height), and underweight (<−2.0 SDs in weight for age) [8,11]. Appropriate weight term was used for all children with z-score weight-for-height values between −2.0 SDs and +2.0 SDs [8].

A severe COVID-19 outcome was defined by the development of clinical acute respiratory distress symptoms (dyspnea, tachypnea, expiratory grunting, nasal flaring), needing supplemental oxygen by a low-flow system for a short period, and/or systemic corticosteroid treatment.

### 2.4. Statistical Analysis

Statistical analysis was performed using Excel 2007 and IBM SPSS v23.0 (Statistical Product and Service Solutions, IBM, New York City, NY, USA). The Shapiro–Wilk test was used to establish the normal data distribution in all groups, and Levene’s test was used for equality of variation. All data were reported as the mean and standard deviation (SD) when normally distributed or as the median and interquartile range (IQR) when non-Gaussian distributed. The primary group data were presented as the number (*n*) and percentage (%). Differences and correlations among groups were tested using the Mann–Whitney U-test or the two-tailed Student’s *t*-test when appropriate.

The logistic regression was used to identify the independent risk factors for SARS-CoV-2 infection and the development of a severe form. The odds ratio (OR) was used to quantify the magnitude of associated risk. The threshold was set at *p* < 0.05 and a 95% confidence interval (95% CI).

## 3. Results

### 3.1. Descriptive Data

A total of 33 children were already admitted to the Pediatric Clinic when the first case of COVID-19 was diagnosed. Sixteen of the 33 children (51.5%) became symptomatic in the next 48 h and tested positive for SARS-CoV-2. A total of 17 children were diagnosed and treated for COVID-19. The median age of all 33 children was 7.0 months (ranging between 2.0 and 24.0 months). The median corrected age calculated according to gestational age was 4.9 months (1.0 to 23.6 months). Both the median age and the median corrected age were higher in the COVID-19-positive group (8.0 months to 7.8 months) compared with the COVID-19 negative group (4.5 months to 3.9 months), but these differences did not reach statistical significance (age: *p* = 0.23; corrected age: *p* = 0.09).

Furthermore, analyzing the birth weight according to gestational age, it was found that the majority of children were AGA (*n* = 20, 60.6%), and 13 (39.4%) had extreme weights (10 SGA and three LGA). The number of children classified as SGA or LGA was more than double in the COVID-19-positive group compared with the COVID-19-negative group (nine vs. four). The most relevant clinical and personal history characteristics in all three groups are presented in Table 1.

As presented in the table above (Table 1), a high percentage of children (72.2%) were considered ‘appropriate weight’ as defined by weight-for-length z-score value. After analyzing the individual-specific growth charts, we observed that 10 of the 24 ‘appropriate weight’ children were, in fact, stunned. Half of the children in this category (*n* = 5) were also underweight. A better nutritional status defined by weight, height, and weight-for-height z-scores was found in COVID-19-negative children compared with positive ones, but the differences were not statistically significant (*p* > 0.05). The main anthropometric characteristics and the differences between groups are presented in Table 2.

### 3.2. Clinical Presentation of COVID-19 in the Study Group

**Figure 1:**
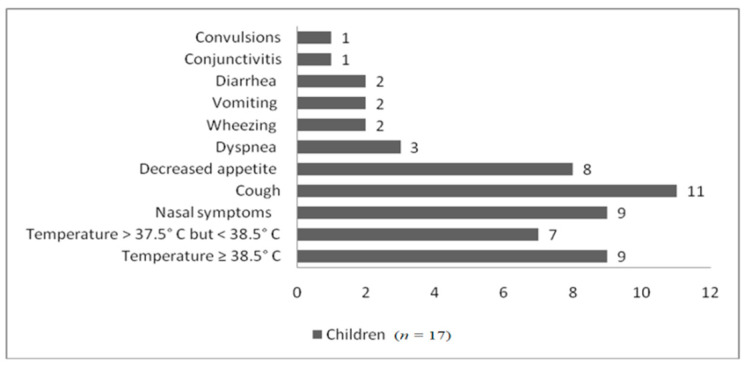
The main symptoms found in SARS-CoV-2-positive children.

All 17 children who tested positive for SARS-CoV-2 experienced respiratory or digestive symptoms. Only one child, already in treatment with anticonvulsant medication (levetiracetam and sodium valproate) for his chronic neurological problems, experienced generalized convulsions as the first sign of infection. Another one had very mild nasal symptoms and conjunctivitis. As shown in Figure 1, the majority of children had mild respiratory symptoms.

Over time, five of the 17 children (29.4%) developed a severe COVID-19 outcome as defined above (see Section 2.3). Three of the five children developed dyspnea, tachypnea, and a positive pulmonary clinical exam. The positive pulmonary clinical exam was defined by the presence of rhonchi, as well as expiratory fine and coarse crackles on auscultation. Supplemental oxygen (the medium FiO_2_ of 4 L/min) and systemic corticoid treatment with intravenous dexamethasone were added to the initial treatment. The other two patients with severe outcomes developed digestive symptoms (vomiting, diarrhea) with moderate dehydration in the first 36 h. Afterward, they experienced expiratory dyspnea, wheezing, and expiratory coarse crackles on auscultation. In addition, they received systemic corticoid treatment, but no supplemental oxygen was needed. None of the children developed acute respiratory distress syndrome (ARDS) or multisystem inflammatory syndrome (MISC-C) in the following months.

The mean duration, in days, until the first SARS-CoV-2-negative test was 10.7 days (minimum 4 days, maximum 15 days). This period was slightly longer in those experiencing a severe form, where the mean was 11.5 days (minimum 8 days and maximum 15 days). The differences between groups were not statistically significant (*p* = 0.46).

### 3.3. Identification of Potential Risk Factors for Severe COVID-19 Outcome

The clinical profiles of children developing a severe form of COVID-19 compared to those with mild and moderate forms are presented in Table 3. After applying logistic regression for each individual risk factor, only a positive pulmonary clinical exam was found to be associated with the risk of severe outcome (OR 2.00; 95% CI, 0.33–5.66; *p* = 0.028) but the results did not reach statistical significance. After adjusting for age, the results were similar, with the OR somewhat higher in children younger than 3 months, but the results still not reach statistical significance.

## 4. Discussion

This study is the first to investigate the clinical risk factors related to the severity of COVID-19 disease in chronic hospitalized young children in our country. The results, along with those from other studies, support the idea that children may have a better short-term prognosis for COVID-19 when compared to adults, even when talking about chronic hospitalized young children with multiple comorbidities [12,13]. Although our study group was formed exclusively from a high-risk population, none of the infants or toddlers developed critical COVID-19 disease or MISC-C.

In addition, we want to draw attention to the importance of malnutrition as a risk factor for developing severe disease. The role of obesity in severe COVID-19 was extensively studied in adults and children [14,15,16]. Around 45% of deaths among children under 5 years are linked to undernutrition, according to WHO [6]. It is well known that chronic diseases can be associated with nutritional problems, and malnutrition was associated with more severe lower respiratory tract infections caused by common pathogens [17]. In a paper about the long-term effects of malnutrition on the severity of COVID-19, it was found that the effect was the highest in children younger than 5 years old and slightly decreased with age [18]. In a global analysis, population-level malnutrition appeared to be related to increased rates of fatal COVID-19 in areas with an elevated burden of undernutrition [19,20]. In our study group, the COVID-19-positive children had a lower mean z-score for weight, height, and weight for height compared with negative children, but these differences did not reach statistical significance. Furthermore, the majority of children (80%, *n* = 4) who developed a severe form of the disease were classified as suffering from malnutrition. Overall, in this study, in line with the data from a recent review, most children who became infected with the SARS-CoV-2 virus had mild respiratory symptoms [21]; the percentage of children developing severe symptoms was higher than in other similar studies (29.4% vs. 7.5%), and all were symptomatic [22]. Malnutrition could play a role, but more extensive studies are needed.

Children with extreme birth weights (SGA, LGA) are at risk for metabolic syndrome, coronary artery disease, osteoporosis, and stroke in later life [23]. However, there are no data regarding the relationship between SGA or LGA and COVID-19 in children. In this study, the number of children with extreme birth weight was two-fold higher in the COVID-19-positive group compared with the negative group (nine vs. four), and four of them developed a severe outcome even if the differences between groups were not statistically significant (*p* = 0.17).

The effect of prematurity was evaluated among children younger than 2 years of age in several studies [24,25]. They concluded that the risk of severe infection was two-fold higher in premature infants than in full-term infants (RR, 2.00; 95% CI, 1.63–2.46) [25]. However, these results were not confirmed by our results, where most premature babies (11 of 18) remained negative and just two children developed severe symptoms.

Male gender, neonatal age group, and pre-existing medical condition were found to be associated with intensive care unit (ICU) admission [6,26]. In the study presented, the majority of infected children were males (M/F: 3.2/1.0), but half of the girls developed a severe outcome. In our relatively small group, the male gender was not a risk factor for severe COVID-19 disease, as also stated in other papers [4,25].

The impact of young age on COVID-19 severity was analyzed in the pediatric population. The majority of authors agreed that there was a lower prevalence of severe illness in children younger than 2 years old, and that younger age (excluding neonates) did not have an impact on the severity of COVID-19 [26,27]. According to a recent meta-analysis, younger age did not have an impact on the severity of COVID-19 (RR, 1.03; 95% CI, 0.74–1.41) [25].

In the same meta-analisys, comorbidities such as congenital heart diseases (RR, 1.82; 95% CI, 1.58–2.09) and neurologic disease, especially seizure (RR, 1.73; 95% CI, 1.43–2.09) but not neurodevelopmental diseases, have been found to be risk factors for severe COVID-19 in children [25]. In the study presented, all five children classified as having severe COVID-19 had congenital heart disease (*n* = 2) or neurologic diseases (*n* = 3), and, in two cases, both pathologies were present. However, the statistical analysis could not demonstrate that cardiac and neurologic underlying pathologies are risk factors for a severe outcome.

Importantly, in this study, the only clinical sign correlated with a severe outcome was a positive pulmonary clinical exam (OR 2.00; 95% CI, 0.33–5.66; *p* = 0.028). Other clinical characteristics such as fever, cough, and dyspnea that were found to be significant risk factors for severe COVID-19 in another meta-analysis analyzing the clinical risk profile in older children [22], could not reach statistical significance in this study.

The children were not wearing face masks during admission or interdisciplinary evaluations. This could be one of the factors influencing our group’s relatively high SARS-CoV-2 infection rate. However, additional studies confirmed that rosacea, irritant contact dermatitis, and acne [28,29] worsened after mask use, even among children [30], consequently reducing the use of face masks. In very young children, data are missing because the Romanian Ministry of Health, in line with WHO recommendations, stated that face masks in children aged 5 years and under are not mandatory [31].

Some important limitations have to behighlighted. Firstly, considering the small number of children and the great heterogeneity of risk factors found in this group, multicenter studies are warranted. Despite these limitations, our findings, in agreement with previous reports, indicate that better outcomes can be found in COVID-19 children compared to adults, even in younger children with previously diagnosed chronic diseases [4,14,15,16,24,25,26,27].

## 5. Conclusions

According to the current state of knowledge, COVID-19 disease in children often has a mild severity even in high-risk populations. The most frequent clinical presentation, even in infants and toddlers, is fever, respiratory symptoms, and problems with feeding. The data are still incomplete, but understanding the risk factors is essential for developing age-specific treatment guidelines, as well as for admission priority criteria, monitoring, and even vaccination.

Malnutrition, including not only the already proven obesity but also undernutrition, seems to negatively influence the clinical course of the disease. The interplay among extreme birth weight, malnutrition, and COVID-19 disease severity underlines the importance of nutritional assessment and intervention across all patient populations, particularly those at risk for COVID-19. In addition, implementing a nutritional screening program in younger children and developing early nutritional intervention strategies may be a positive outcome from this pandemic.

The classic clinical pulmonary exam remains a reliable screening tool in evaluating these patients, but further studies are needed to confirm these results using more expensive but standardized methods (pulmonary ultrasound or chest radiographs and even computer tomography).

## Figures and Tables

**Table 1:** The clinical and personal history features distribution for the studied groups.

Characteristics	Participants, *n* (%)			
	COVID-19 Positive	COVID-19 Negative	All	*p*-Value
	17	16	33	
Sex:				
Male	13 (76.5)	7 (43.7)	20 (60.6)	0.052
Female	4 (23.5)	9 (56.3)	13 (39.4)	(ref)
Age:				
0–3 months	3 (17.6)	5 (31.2)	8 (24.2)	0.36
4–12 months	7 (41.2)	8 (50.0)	15 (45.5)	0.61
>12 months	7 (41.2)	3 (18.8)	10 (30.3)	0.15
*Comorbidities:*				
Prematurity *	7 (41.2)	11(68.8)	18 (54.5)	0.10
*Nutritional status:*				
Appropriate weight	10 (58.8)	14 (87.5)	24 (72.2)	0.11
Stunning	9 (52.9)	5 (31.2)	14 (42.4)	0.20
Wasting	8 (47.0)	3 (18.8)	11 (33.3)	0.12
Underweight	6 (35.3)	2 (18.8)	8 (24.2)	0.08
*Personal history of:*				
Neurodevelopmental disorders	9 (52.9)	9 (56.2)	18 (54.5)	0.84
Cardiac or circulatory congenital anomalies	7 (41.2)	5 (31.2)	12 (36.4)	0.55
Gastrointestinal anomalies including surgical correction	3 (17.6)	5 (31.2)	8 (24.2)	0.36
Pulmonary diseases	1 (5.9)	0 (0.0)	1(3.0)	-
Genetic Syndromes	1 (5.9)	2 (12.5)	3 (9.1)	-
Perinatal exposure to maternal infectious diseases **	2 (11.8)	0 (0.0)	2 (6.1)	-
Others ***	3 (17.6)	7 (43.7)	10 (30.0)	0.100
*Vaccination status:*				
BCG	3 (17.6)	5 (31.2)	8 (24.2)	0.361
Anti VHB	7 (41.2)	6 (37.5)	13 (39.4)	0.892
Hexavalent + P13(1st dose)	10 (58.8)	10 (62.5)	20 (60.6)	0.892
Hexavalent + P13(2nd dose)	6 (35.3)	3 (18.7)	9 (27.3)	0.282

* Prematurity: gestational age ranging between 25 weeks and 36 weeks and 5 days. BCG: Bacillus Calmette–Guerin vaccine; VHB: virus hepatitis B; P13: pneumococcal polysaccharide conjugate vaccine (13-valent, adsorbed). ** Perinatal exposure to HIV or VHB; *** one child had a renal malformation, while the remainderhad anemia.

**Table 2:** Anthropometric measurements of the study group according to SARS-CoV-2 infection status.

Characteristics	Median (Range)			
	COVID-19 Positive	COVID-19 Negative	All	*p*-Value
	17	16	33	
Weight (kg)	7.00 (3.40–9.30)	5.65 (4.00–10.50)	6.20 (3.40–10.5)	0.12
Weight z-score	−1.92 (−4.52–+0.70)	−0.91 (−2.62–+1.92)	−1.23 (−4.52–+1.92)	0.06
Height (cm)	68 (52–89)	59.5 (50–90)	61 (50–90)	0.22
Height z-score	−2.02 (−5.89–+1.53)	−0.90 (−3.48–+1.72)	−1.22 (−5.89–+1.72)	0.15
Weight-for-height z-score	−1.29 (−4.11–+2.36)	−0.33 (−3.64–+1.60)	−0.44 (−4.11–+2.36)	0.36

**Table 3:** Identification of risk factors for severe outcome in COVID-19 children (mild vs. severe outcome).

Characteristics	Participants (*n* = 17)		
	COVID-19 Mild/Moderate	COVID-19 Severe	*p*-Value
	*n* (%)	*n* (%)	
	*n* = 12	*n* = 5	
Sex			
Male	10 (58.8)	3 (17.6)	0.31
Female	2 (11.8)	2 (11.8)	(ref)
Age			
0–3 months	2 (11.8)	1 (5.9)	0.87
>3 months	10	4	(ref)
Extreme birth weight (LGA/SGA)	5	4	0.17
Prematurity	5	2	0.94
Nutritional status			
Malnutrition	8 (47.1)	4 (23.5)	0.57
Stunning	4	4	0.13
Wasting	6	1	0.38
Underweight	5	2	0.70
**Comorbidities**			
Neurodevelopmental disorders	5	3	-
Cardiac or circulatory congenital anomalies	4	2	-
Gastrointestinal anomalies including surgical correction history	2	1	-
Anemia	1	2	-
Positive personal history *	8 (47.1)	5 (29.4)	0.82
**Clinical signs**			
Fever	7	2	0.70
Temperature	4	3	0.31
Cough	7	4	0.38
Positive pulmonary clinical exam	2 (11.8)	4 (23.5)	**0.02**
Digestive symptoms	1	2	0.13

Percentages are calculated from the whole group (*n* = 17); * positive personal history for neurodevelopmental disorders and congenital heart diseases.

## Data Availability

Data are contained within the article. The raw data are not publicly available due to privacy reasons.
